# Endoscopic findings in uninvestigated dyspepsia

**DOI:** 10.1186/1471-230X-14-19

**Published:** 2014-02-06

**Authors:** Jacob Jehuda Faintuch, Fernando Marcuz Silva, Tomás Navarro-Rodriguez, Ricardo Correa Barbuti, Claudio Lyoiti Hashimoto, Alessandra Rita Asayama Lopes Rossini, Marcio Augusto Diniz, Jaime Natan Eisig

**Affiliations:** 1 Division of Clinical Medicine and Propaedeutic Clinic of Hospital das Clínicas da Faculdade de Medicina da USP, São Paulo, Brasil; 2 Division of Gastroenterology and Clinical Hepatology of Hospital das Clínicas da Faculdade de Medicina da USP, São Paulo, Brasil; 3 Hospital das Clínicas da Faculdade de Medicina da Universidade de São Paulo, Av. Dr. Enéas de Carvalho Aguiar, 155 – Cerqueira César, São Paulo, SP, Brasil

**Keywords:** Dyspepsia findings, Esophagitis, Functional dyspepsia, Gastric cancer, Peptic ulcer

## Abstract

**Background:**

It is important to know the causes of dyspepsia to establish the therapeutic approach. Dyspepsia is a frequent syndrome in our country, where there are restrictions to endoscopy and high prevalence of *Helicobacter pylori (H. pylori)* infection. This study aimed to assess the endoscopic findings of the syndrome, in an outpatient screening clinic of a tertiary hospital in São Paulo.

**Methods:**

Outpatients with uninvestigated dyspepsia, according to Rome III criteria, answered a dyspepsia questionnaire and underwent esophagogastroduodenoscopy. The Rapid Urease Test was applied to fragments of the antral mucosa and epidemiological data were collected from the studied population. Organic dyspepsia findings were analyzed with different variables to verify statistically significant associations.

**Results:**

Three hundred and six patients were included and 282 were analyzed in the study. The mean age was 44 years and women comprised 65% of the sample. Forty-five percent of the patients reported alarm symptoms. Functional dyspepsia was found in 66% of the patients (20% with normal endoscopy results and 46% with gastritis), 18% had GERD and 13% had ulcers (duodenal in 9% and gastric in 4%). Four cases of gastric adenocarcinoma were identified (1.4%), one without alarm characteristics, 1 case of adenocarcinoma of the distal esophagus and 1 case of gastric lymphoma. The prevalence of *H. pylori* was 54% and infection, age and smoking status were associated with organic dyspepsia. The age of 48 years was indicative of alarm signs.

**Conclusions:**

The endoscopic diagnosis of uninvestigated dyspepsia in our setting showed a predominance of functional disease, whereas cancer was an uncommon finding, despite the high prevalence of *H. pylori*. Organic dyspepsia was associated with infection, age and smoking status.

## Background

Dyspepsia is a prevalent complaint in general practice and gastrointestinal clinics [1-5], with a prevalence of up to 40% among adults in Brazil [6]. Dyspepsia represents up to 8.3% of all primary care physician visits and causes huge economic costs to patients and to the economy [7]. Rome III guideline states that dyspepsia is non-reflux predominant pain or discomfort in the upper abdomen and the patients must also have one or more of the following four symptoms: postprandial fullness, early satiation, epigastralgia and epigastric burning. Symptom onset must have occurred at least six months prior to diagnosis [8]. Only 75% of the dyspepsia experts, 73% of gastroenterologists and 59% of primary care providers adhere to dyspepsia best practices; so “dyspepsia” means different things to different providers. Without a common diagnostic language, general practitioners may be unable to provide adequate treatment following common dyspepsia guidelines. [9]. The rapid introduction of new diagnostic criteria for dyspepsia has made very difficult or virtually impossible to compare prevalence rates from different periods or geographic regions [10]. Because structural upper gastrointestinal (UGI) tract diseases, such as peptic ulcer, erosive esophagitis, luminal strictures and malignancy can course with dyspepsia, esophagogastroduodenoscopy (EGD) is the diagnostic procedure of choice to differentiate patients with organic from those with functional dyspepsia [11]. Although it is possible to propose endoscopy as the initial strategy for dyspepsia [12], the establishment of this procedure for every dyspeptic patient may not be practical approach, as the high prevalence of the syndrome will result in very high costs to any health system [13]. Moreover, the diagnostic procedure and its cost effectiveness must be considering that a large number of uninvestigated dyspepsia are functional cases [14]. Thus, the use of endoscopy in the management of uninvestigated dyspepsia remains a controversial issue worldwide [11]. The frequency of uninvestigated dyspepsia varies considerably in different populations and such differences may be related to true differences in the frequency of the condition or the criteria used to diagnose dyspepsia [15]. International medical practice and academic associations have recommended using alarm signs with or without age limits, usually set at 50–55 years, to select dyspeptic patients for endoscopy [16]. The predictive values to be used in the diagnosis of upper gastrointestinal pathology have been extensively studied, but the results are inconsistent, especially because the majority of previous studies were carried out in Europe or North America [16-18]. As for our country, the very high prevalence of *H. pylori* infection [19-22], which requires a complex and expensive treatment for a large number of individuals and the low availability of noninvasive tests for the diagnosis of *H. pylori* infection make the test and treat approach unfeasible. The age indication for endoscopy has not been determined in our country and the limited availability of this procedure does not allow it to be requested as the initial approach.

By prospectively following consecutive patients with uninvestigated dyspepsia in an outpatient screening clinic from a tertiary hospital, this study aimed to assess the diagnostic effectiveness of EGD, in a developing country.

## Methods

### Study patients and setting

This prospective observational study was carried out in a tertiary hospital, which provides open-access service to endoscopy. From September 2008 and September 2011, consecutive adult outpatients who presented with uninvestigated dyspepsia were screened for eligibility. All study participants were systematically evaluated before undergoing endoscopy. The patients were interviewed to determine the presence of alarm symptoms, including unintended weight loss (defined as decrease of more than 5% of original body weight in three months), symptoms suggestive of upper gastrointestinal bleeding and dysphagia. Older age, presence of mass or lymphadenopathy and family history of upper gastrointestinal cancer were not included as alarm characteristics. Symptom intensity was determinate by the Leeds Dyspepsia Questionnaire [23] and epigastralgia was considered typical when pain was relieved by food or acid suppression or clocking was present. The present study was carried out by only two physicians, who made the interviews in person with the outpatients using a standardized questionnaire. The upper digestive endoscopy was carried out with a standard electronic videoendoscope by two experienced endoscopists, no later than 20 days after the interview, to allow time for the symptomatic use of antacids. *H. pylori* determination was performed by the Rapid Urease Test, validated in our country [24].

### Inclusion criteria

Epigastralgia or epigastric burning lasting for at least three months, with symptom onset having occurred at least six months before, at least once a week and/or at post-prandial fullness or early satiation, for three months, with symptom onset that started at least six months before, at least once a week. Patients should be younger than 90 and older than 13 years old.

### Exclusion criteria

Exclusion criteria included predominant symptoms of gastroesophageal reflux disease (GERD), symptoms outside the epigastrium, other predominant dysmotility symptoms (nausea and vomiting), use of NSAIDs (including low dose treatment) up to one week before study inclusion, use of proton pump inhibitors or H2-blockers for more than two weeks, before study enrollment, presence of systemic decompensated disease (congestive heart failure, coronary heart disease, liver failure, diabetes mellitus, thyroid disease, acute or chronic respiratory failure, hematological diseases), presence of major psychiatric disorders, impediment to endoscopy and difficulty for the patient to understand the aims and procedures of the study.

### Ethics

This study was approved by the Ethics Committee for Analysis of Research Projects - CAPPesq - Clinical Direction of the Hospital and the Faculty of Medicine, University of São Paulo. Written informed consent was obtained from the patients prior to study participation.

### Statistical analysis

Variables were measured as frequency and percentage and the association between organic dyspeptic findings and the variables was determined by Fisher’s test, with a p value < 0.05 being considered statistically significant. A cutoff for age was obtained though ROC curve.

Organic dyspeptic findings were analyzed with the variables by simple and multiple binary logistic regressions then odd ratios and its 95% confidence intervals were presented. A score for endoscopy indication was determined based on regression coefficient values. Calculations were performed using R (The R Foundation for Statistical Computing), version 2.15.2.

## Results

Three hundred six patients were included, of which 282 were available for analysis: 8 patients dropped from the study, 5 were excluded, as they were using of omeprazole at the time of the endoscopy, 4 had their endoscopy results lost, 3 refused to undergo endoscopy, 3 underwent the endoscopy after more than 30 days and one had a previous abdominal surgery.

Patient demographic data are shown in Table 1 and symptoms are listed in Table 2.

**Table 1:** Demographic informations of patients studied

Patients included	306
Endoscopies performed	282 (92%)
Positive *H. pylori*	144 (54%)
Age-mean years	44
Range	16 – 87
Age > 48	121 (42%)
Male gender	98 (35%)
Race- white	203 (72%)
Born in São Paulo	120 (43%)
Smokers	45 (16%)

**Table 2:** Description of the symptoms

Symptoms onset	
6 to 11 months	144 (51%)
1 to 3 years	75 (26%)
4 to 10 years	35 (12%)
More than 10 years	28 (10%)
Type of symptoms	
Epigastralgia	193 (68%)
Post-prandial fullness	89 (32%)
Intensity of symptoms	
Mild	62 (22%)
Moderate	125 (44%)
Severe	94 (33%)
Pain specificity	
Tipic	113/192 (59%)
Atipic	79 (41%)
Alarm symptoms	
Weight loss	101 (36%)
Bleeding	14 (5%)
Dysphagia	11 (4%)

GERD (18%) and peptic ulcer (13%) were the major causes of organic dyspepsia; there were six cases (2%) of upper gastrointestinal cancer (4 gastric carcinomas, 1 gastric lymphoma and 1 esophageal adenocarcinoma) characterizing a total of 96 (34%) patients as having organic dyspepsia (Table 3). Reflux disease included cases of erosive esophagitis, Barrett’s esophagus and esophageal ulcer. The specific findings on upper digestive endoscopy are summarized in Table 4. Organic dyspepsia (determined by the finding of reflux disease or peptic ulcer or malignancy) showed statistical significance with age, *H. pylori* positive status and smoking, but not with weight loss, symptom intensity and duration, gender and ethnicity (Table 5).

**Table 3:** General endoscopic findings

Functional dyspepsia	186 (66%)
Normal examen	56 (20%)
Gastritis	130 (46%)
Organic dyspepsia	96 (34%)
Reflux esophagitis	52 (18%)
Peptic ulcer	38 (13%)
Gastric	12 (4%)
Duodenal	26 (9%)
Malignancy	6 (2%)
Gastric adenocarcinoma	4 (1.4%)
Gastric lymphoma	1 (0.4%)
Esophageal adenocarcinoma	1 (0.4%)

**Table 4:** Specific endoscopic findings

Esophagus	
Non-erosive esophagitis*	8 (3%)
Erosive esophagitis	47 (16%)
Barret	2 (0.7%)
Ulcer	1 (0.4%)
Others**	10 (4%)
Stomach	
Gastritis	186 (61%)
Enanthematous	66 (23%)
Erosive	95 (34%)
Nodular	18 (6%)
Atrophic	7 (3%)
Ulcer 14 (5%)	
Fundus, body and antrum	9 (3%)
Prepyloric	5 (2%)
Malignancy	5 (1.8%)
Adenocarcinoma	4 (1.4%)
Lymphoma	1 (0.4%)
Others	6 (2%)
Duodenum	
Duodenitis	31 (11%)
Enanthematous	9 (3%)
Erosive	22 (8%)
Ulcer	6 (9%)
Others	10 (4%)

*Non erosive esophagitis: enanthematous esophagitis: 6 cases, esophageal candidiasis: 2 cases.

**Others esophagus findings: Hiatal hernia: 6 cases, Papillomatosis esophagitis: 2 cases, Esophageal variz: 1 case, Retention cyst: 1 case.

**Table 5:** Organic dyspepsia in simple binary logistic regression with variables

***Variable***	***Group***	***OR***	***95% CI***	***p Value***
Gender	Male	1.54	0.92 – 2.56	0.10
Age	> 48	1.75	1.06 – 2.87	0.03
Smoking	Positive	1.4	1.21 – 4.4	0.01
Weight loss	Positive	0.7	0.42 – 1.18	0.18
Symptom Intensity	Severe	0.56	0.28 – 1.11	0.10
Symptom duration	> 10 years	0.67	0.28 - 1,62	0.37
*H. pylori*	Positive	1.67	1.06 – 2.87	0.05

**Figure 1:**
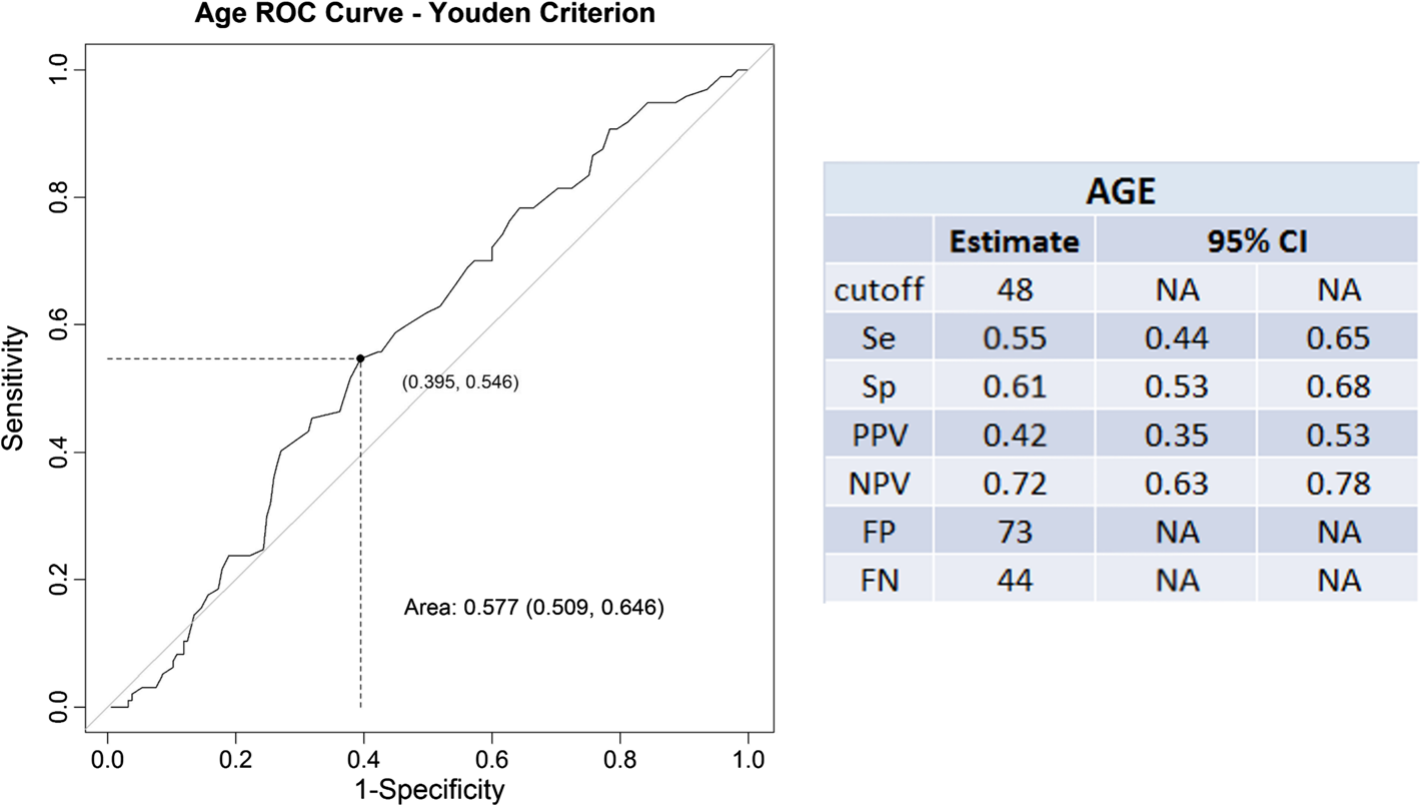
Age indication for alarm feature.

The age of patients analyzed by ROC curve showed that the age of 48 years had a 0.42 positive and 0.72 negative predictive values for organic dyspepsia (Figure 1), suggesting this age is an alarm characteristic.

**Figure 2:**
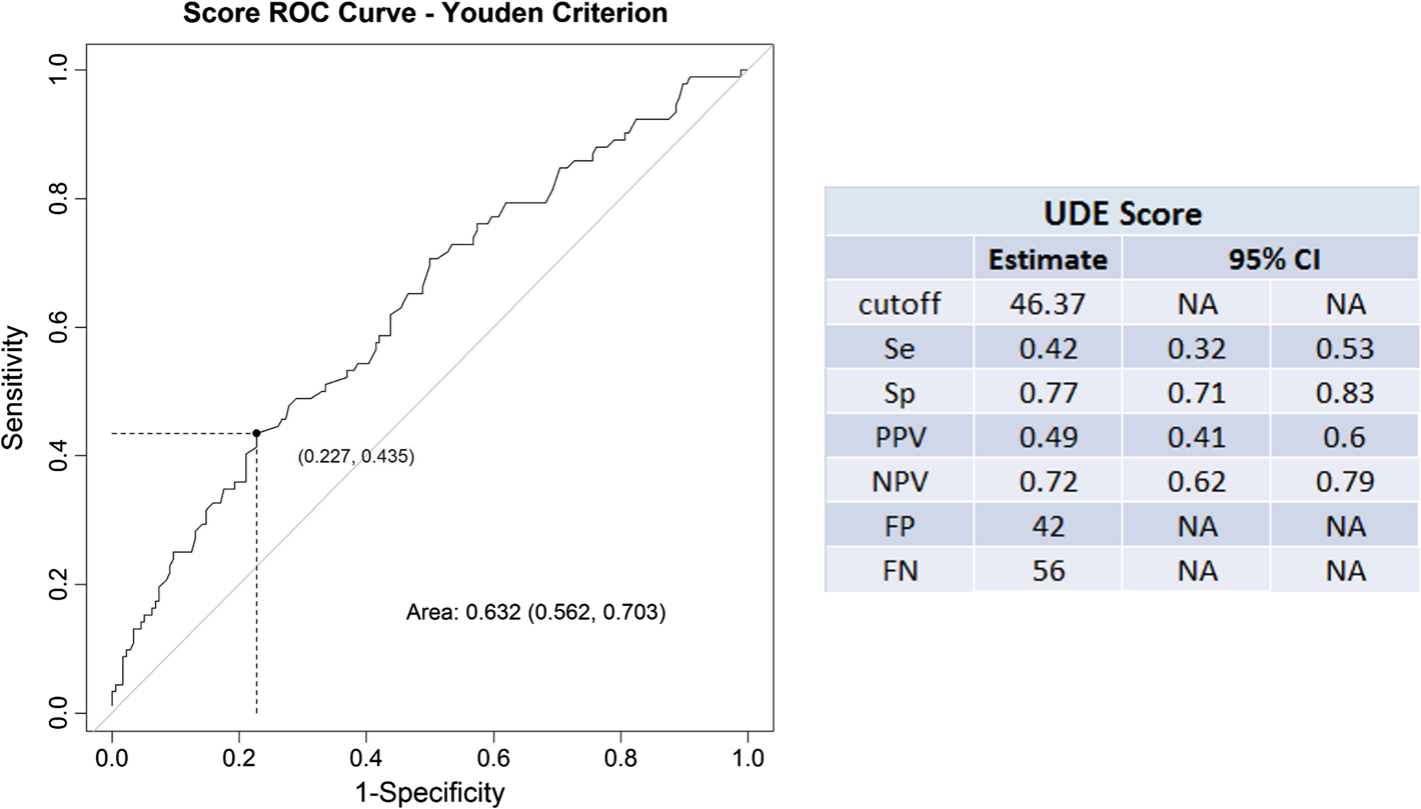
Upper Digestive Endoscopy (UDE) Score.

The coefficients of multiple binary logistic regression of age, treated as a continuous variable, smoking status and positive *H. pylori* status (Table 6) allowed the construction of a score, where values lower than 46 (in a scale of up to 100 points) indicates the non- necessity of EGD with a high negative predictive value for organic dyspepsia (Figure 2).

**Table 6:** Organic dyspepsia in multiple binary logistic regression

***Variable***	***Group***	***OR***	***95% CI***	***pValue***
Age	> 48	1.92	1.13 – 3.25	0.02
Smocking	Positive	2.36	1.19 – 4.69	0.01
*H. pylori*	Positive	1.68	0.99 – 2.86	0.05

## Discussion

Our study shows results that are consistent with the meta-analysis by Ford [14], although (considering the Rome criteria) our prevalence of GERD was somewhat higher than that of peptic ulcer, whereas malignancy rates were somewhat higher than those observed in that study. These differences may be due to the fact that our institution is an outpatient screening clinic in general practice of a tertiary hospital. In patients followed in a three-year prospective general practice study, the presence of alarm symptoms significantly increased the risk of developing peptic ulcers, but not gastrointestinal cancer. Positive predictive values for development of cancer and ulcer were 4% and 14%, respectively [18]. Patients with peptic ulcer were more likely to present with gastrointestinal bleeding [25] and in our study, gastrointestinal bleeding was an uncommon alarm symptom (5%), whereas the prevalence of peptic ulcer was 13% and malignancy, 2%. It was somewhat surprising that more than two fifths of our functional dyspeptic patients had alarm symptoms, while about 75% of the ulcer patients did not. It is possible that our most frequent alarm symptom (weight loss) was not specific for serious digestive tract diseases.

Upper GI bleeding and unintended weight loss were also associated with malignancy [26], but the sensitivity of alarm features in diagnosing upper gastrointestinal malignancy varied from 0% to 100%, while specificity ranged from 16% to 98% [27]. This wide variation in sensitivity may be due to the small number of cancer cases detected in many of the studies. Five in six patients with neoplasms had alarm symptoms, inferring a sensitivity of 83% and specificity of 59%. In a study with unsedated transnasal esophagogastroduodenoscopy, cancer was found in 4% of patients with alarm symptoms (in our study 1.7%) *versus* 0.1% (in our study 0.6%) in the non-alarm symptom group [27]. Despite the difference between patients with and without alarm symptoms, it is known that symptoms have limited value in the diagnosis of upper gastrointestinal malignancy [28].

In this study, older age, mass or lymphadenopathy and family history of upper gastrointestinal cancer were not included as alarm features. In Brazil there is no consensus on this matter and the AGA guidelines are usually followed [16]. In our sample, all patients with malignancy were older than 55 years, but considering the finding of organic dyspepsia (reflux disease, peptic ulcer and malignancy) our study suggests the age of 48 as indicative of alarm symptom.

Frequent vomiting was not considered an alarm symptom, as it was disregarded when reported as a chief complaint in dyspeptic syndrome and thus, it is unlikely that this symptom, when present for at least three months, will not result in weight loss.

The presence of adenopathy or abdominal tumor changes the diagnosis of undiagnosed dyspepsia into undiagnosed adenopathy or tumor and in these cases, the best approach requires imaging assessment and not an esophagogastroduodenoscopy.

Family history of upper gastrointestinal cancer is a type of information that is difficult to obtain, when patients know the cause of the disease, they cannot provide information on its type and precise location.

Primary gastrointestinal lymphoma is a rare disease, although the stomach is the most frequent site of involvement for this neoplasm [29]. Our sample had only 1 case of lymphoma; considering the small sample size of our study, this finding was most likely fortuitous.

The prevalence of GERD has increased dramatically in recent decades, mostly in the western world, where it affects about 19% to 30% of the population, increasing the risk for esophageal adenocarcinoma [30]. In this study, GERD was diagnosed in 18% of patients, similar to the findings of a recent meta-analysis, based on Rome criteria [14]. In Denmark, gastric inflammation was recently found in 11% of the patients with upper gastrointestinal symptoms [27]; our study did not include histological examination of the gastric mucosa, and thus, gastritis was an endoscopic diagnosis, which after the exclusion of other concurrent diagnoses showing a prevalence of 46%. The prevalence of *H. pylori* infection in our population was high (54%) and infected individuals had a 10-fold higher probability of having any gastric mucosa lesions than non-infected individuals [31]. Our finding of 38 patients (13%) with ulcer, 26 of them (9%) with duodenal ulcer, is also consistent with the high prevalence of infection.

In 2005, in a study of our hospital, the overall *H. pylori* prevalence was 53%, assessed in 1478 consecutive endoscopies [32]. We had a similarly high prevalence, indicating that there was no decrease in infection rates during this period.

This high prevalence of infection associated with the low availability of non-invasive tests for its detection prevent the use of the proposed approach of test and treat strategy for undiagnosed dyspepsia. *H. pylori* eradication treatment is always high cost and complex, with limited efficiency of 88% [33]. The number of cases of functional dyspepsia responsive to treatment is low [34], as only 50% of ulcer patients attain symptom resolution [35,36], whereas the symptoms of patients with reflux disease do not improve with treatment [37]. Therefore, the test and treat strategy may not be adequate for developing countries, which usually have very high prevalence of *H. pylori* infection and low level of resources for health care. Empirical treatment for young patients without alarm signs may be the possible approach for undiagnosed dyspepsia in our country.

Our study provides a score, which can be useful in indicating endoscopy for these cases. Further studies should be performed to test this proposal, especially in centers similar to ours.

## Conclusions

In Brazil, a developing country with high prevalence of *H. pylori* infection, the most frequent cause of uninvestigated dyspepsia is functional dyspepsia, whereas gastric cancer is a rare finding. Even after selecting patients according to Rome III criteria, reflux disease predominated over peptic ulcer.

The suggested age for the onset of alarm signs is 48 years old and a score that correlates *H pylori* infection, age and smoking may be useful for the indication of endoscopy as the approach in the presence of dyspepsia.

## Competing interests

The authors declare that they have no competing interests.

## Authors’ contributions

All authors contributed to the design of the study. Acquisition of data and quality control: JJF, FMS. Analysis and interpretation of data: JJF, FMS, TNR, RCB,MAD, JNE. Endoscopic examinations: CLH, ARALR. Statistical Analysis: MAD. Draft manuscript: JJF. All authors have read and approved the final manuscript.

## Pre-publication history

The pre-publication history for this paper can be accessed here:

http://www.biomedcentral.com/1471-230X/14/19/prepub
